# Network Analysis of Different Exogenous Hormones on the Regulation of Deep Sowing Tolerance in Maize Seedlings

**DOI:** 10.3389/fpls.2021.739101

**Published:** 2021-12-02

**Authors:** Fenqi Chen, Xiangzhuo Ji, Mingxing Bai, Zelong Zhuang, Yunling Peng

**Affiliations:** Gansu Provincial Key Laboratory of Aridland Crop Science, Gansu Agricultural University, College of Agronomy, Gansu Agricultural University, Gansu Key Lab of Crop Improvement & Germplasm Enhancement, Lanzhou, China

**Keywords:** maize, deep seeding, transcriptome analysis, WGCNA, different hormones, network regulation

## Abstract

The planting method of deep sowing can make the seeds make full use of water in deep soil, which is considered to be an effective way to respond to drought stress. However, deep sowing will affect the growth and development of maize (*Zea mays* L.) at seedling stage. To better understand the response of maize to deep sowing stress and the mechanism of exogenous hormones [Gibberellin (GA_3_), Brassinolide (BR), Strigolactone (SL)] alleviates the damaging effects of deep-sowing stress, the physiological and transcriptome expression profiles of seedlings of deep sowing sensitive inbred line Zi330 and the deep-tolerant inbred line Qi319 were compared under deep sowing stress and the conditions of exogenous hormones alleviates stress. The results showed that mesocotyl elongated significantly after both deep sowing stress and application of exogenous hormones, and its elongation was mainly through elongation and expansion of cell volume. Hormone assays revealed no significant changes in zeatin (ZT) content of the mesocotyl after deep sowing and exogenous hormone application. The endogenous GA_3_ and auxin (IAA) contents in the mesocotyl of the two inbred lines increased significantly after the addition of exogenous GA_3_, BR, and SL under deep sowing stress compared to deep sowing stress, while BR and SL decreased significantly. Transcriptome analysis showed that the deep seeding stress was alleviated by GA_3_, BR, and SLs, the differentially expressed genes (DEGs) mainly included cellulose synthase, expansin and glucanase, oxidase, lignin biosynthesis genes and so on. We also found that protein phosphatase 2C and GA receptor GID1 enhanced the ability of resist deep seeding stress in maize by participating in the abscisic acid (ABA) and the GA signaling pathway, respectively. In addition, we identified two gene modules that were significantly related to mesocotyl elongation, and identified some hub genes that were significantly related to mesocotyl elongation by WGCNA analysis. These genes were mainly involved in transcription regulation, hydrolase activity, protein binding and plasma membrane. Our results from this study may provide theoretical basis for determining the maize deep seeding tolerance and the mechanism by which exogenous hormones regulates deep seeding tolerance.

## Introduction

Maize (*Zea Mays* L.) is the most widely grown and important food, feed, and industrial feedstock crop in the world (Niu et al., 2019a). A disadvantage of maize is that it is sensitive to a variety of abiotic stresses (such as salt stress, drought stress, and low temperature stress) during its seedling life (Chen et al., 2020). In China, maize planting areas are mostly distributed in arid and semi-arid areas, accounting for 2/3 of cultivated land (Du et al., 2017). The threat of drought to maize production is unpredictable (Daryanto et al., 2016).

While the area under maize cultivation in China has been gradually expanding over the past 10 years, the population has also grown, increasing the total food demand. It is expected that in 10 years, the population base of China will reach 1.5 billion, and the total food demand will reach 650 million tons (Xiang and Zhong, 2013). Rapid economic and population growth in China has led to increasing water demand for agriculture and domestic uses, making water resources increasingly scarce and thus threatening sustainable agricultural development (Li et al., 2017). Important production areas for corn include the northeast, north, and northwest, which are also the main arid and semi-arid regions in China. Relying mainly on natural rainfall, they face problems such as dry spring seasons with little rainfall, low soil moisture content, and no irrigation conditions. As a result, drought has seriously hampered the improvement of maize production in these areas (Zhao G. W. et al., 2009b; Li et al., 2016).

Deep sowing is an effective way to reduce the impacts of drought stress, and is widely used in rice (*Oryza sativa* L.) planting, which is superior to the normal sowing as it protects the rice seedlings from the damage of drought stress. Importantly, deep-sown rice matures earlier and has higher yield under drought conditions than transplanted rice (Cao et al., 2005). Thus, in the face of the continuous expansion of corn cultivation area, selection of deep-sowing tolerant maize varieties is becoming increasingly important in arid and semi-arid regions (Niu et al., 2019a). However, when crop seeds are sown deeply, the emergence time of germinating seeds is often prolonged, the emergence rate is reduced, and seedling vigor is diminished because of increased soil resistance (Rebetzke et al., 2007).

It has previously been shown that crops with deep-sowing tolerance traits generally have more developed elongated organs that give the main impetus for their plumule to expose the soil surface. Deep sowing tolerance in rice and maize is closely related to elongation of the mesocotyl (Zhao et al., 2018; Niu et al., 2019b). Because of the mesocotyl rapid elongation during seed germination, which pushes the germinal sheath (and internal germ) out of the soil surface, promoting seed emergence (Lee et al., 2012; Kutschera and Wang, 2016). Previous attempts to circumvent the problems associated with deep sowing included application of growth hormones such as abscisic acid (ABA), IAA, GA_3_, and ethylene (ETH). The sensitivity of maize mesocotyl elongation to GA_3_ was higher at 20 cm deep sowing than at 2 cm sowing depth (Zhao and Wang, 2008), and was mainly increased cell elongation rather than cell division (Zhao and Wang, 2010). In rice, Watanabe and Takahashi (1999) showed that ABA, GA_3_, IAA, and ETH all promoted mesocotyl elongation, with GA_3_, IAA, and ETH promoting cell elongation, and ABA promoting cell division. Kutschera and Wang (2016) found that maize isolated mesocotyls responded more rapidly to exogenous IAA. After exogenous IAA treatment, maize isolated mesocotyls significantly elongated, and the cell elongation was mainly achieved through the acidification and relaxation of mesocotyl cell wall. The regulation mechanism of exogenous BR was consistent with that of IAA, and the two hormones cooperate to regulate the elongation of mesocotyl (Deng et al., 2012). Cytokinin (CTK) has also been shown to promote mesocotyl elongation through cell division in rice (Cao et al., 2005). On the contrary, SL negatively regulates mesocotyl growth by inhibiting cell division but not cell elongation in rice in darkness (Hu et al., 2010). However, there are no reports on the study of mesocotyl elongation under deep seeding stress in maize. Therefore, identifying the determinants affecting mesocotyl elongation will be helpful for selecting crop varieties that are tolerant of deep sowing.

The physiological responses and molecular mechanisms regarding exogenous BR, SL, and GA_3_ to alleviate deep sowing stress in maize are unclear. Therefore, these three hormones were used to treat deep-sowing-sensitive and deep sowing tolerant inbred lines under deep sowing stress by root irrigation. The transcriptional expression profiles of maize inbred lines with different deep sowing tolerance under deep sowing stress were analyzed by comparative transcriptomic approaches after the application of exogenous BR, SL, and GA_3_ to investigate the mechanisms of deep sowing tolerance, mesocotyl elongation and the mechanisms of exogenous hormone regulation of deep sowing tolerance in maize and the screening of relevant candidate genes.

## Materials and Methods

### Experimental Materials

According to the preliminary laboratory test basis, the deep sowing tolerant maize autotrophic line Qi319 and the deep sowing sensitive autotrophic line Zi330, which differed significantly in mesocotyl length under deep sowing stress, were selected for deep sowing stress and exogenous substances to alleviate deep sowing stress. The exogenous substances were GA_3_ (Solarbio, Beijing, China), BR (Macklin, Shanghai, China), SL (Solarbio, Beijing, China).

### Material Handling

The seeds were first sterilized with 0.5% NaClO solution for 10 min, rinsed 3–5 times with ddH_2_O, and soaked in distilled water for 12 h. After water was blotted out with sterilized filter paper, the seeds were sown in PVC tubes (17 cm in diameter and 50 cm in height) filled with sterilized vermiculite and distilled water in the ratio of 5 g:1 mL, and the bottom of the tubes were sealed with nylon mesh. Ten seeds were sown per PVC tube. Seeds were covered with 3 cm of soil for the normal sown control and 15 cm of soil for the deep sowing stress. The light incubator conditions were 60% relative humidity, 25 and 22°C during day and night, respectively, with a 14/10 h light/dark cycle, and 600 μmol⋅s^–1^⋅m^–2^ light intensity. Measurements of indices related to deep sowing tolerance were made 12 days after germination. BR treatment concentrations: 3 cm + 0 mg⋅L^–1^ BR (CK), 15 cm + 0 mg⋅L^–1^ BR (DS), 3 cm + 0.4 mg⋅L^–1^ BR (CKBR), 15 cm + 0.4 mg⋅L^–1^ BR (DSBR), SL treatment concentrations: 3 cm + 0 mg⋅L^–1^ SL (CK), 15 cm + 0 mg⋅L^–1^ SL (DS), 3 cm + 0.5 μmol⋅L^–1^ SL (CKSL), 15 cm + 0.5 μmol⋅L^–1^ SL (DSSL), GA treatment concentrations: 3 cm + 0 mg⋅L^–1^ GA_3_ (CK), 15 cm + 0 mg⋅L^–1^ GA_3_ (DS), 3 cm + 4.0 mg⋅L^–1^ GA_3_ (CKGA), 15 cm + 4.0 mg⋅L^–1^ GA_3_ (DSGA). In addition, the concentration of exogenous hormone treatment was the best concentration selected in the early stage. Three biological replicates were set for each treatment, and 50 ml distilled water or corresponding treatment solution was applied every 2 days after sowing.

### Determination of Related Indexes of Deep Sowing

Mesocotyl length (MES), coleoptile length (COL), seedling length (SDL), and root length (RL) were measured with scale and analytical balance after 12 days of seed germination. The paraffin section technique (as described by Schuerger et al., 1997) with safranin-fixed green staining was used to observe the histological structure of the middle part of maize mesocotyl.

The relative contents of zeatin (ZT), IAA, and GA_3_ were determined in maize mesocotyl by high performance liquid chromatography (HPLC) as described by Zhao et al. (2021). The relative contents of endogenous BR and SL were determined by HPLC tandem mass spectrometry (HPLC-MS/MS) as described by Ghosh et al. (2019).

### Transcriptional Analysis

#### Total RNA Extraction and Library Construction

Mesocotyl samples of individual maize materials were frozen at –80°C in an ultra-low temperature refrigerator, and the RNA from the total samples was isolated and purified according to the protocol provided by the TRIzol (Invitrogen, CA, United States) reagent manufacturer. The amount and purity of the extracted total RNA was then quality controlled using NanoDrop ND-1000 (NanoDrop, Wilmington, DE, United States). The total RNA of each treated sample was taken for the construction of the RNA-seq library, which was done by Lianchuan Bio (Hangzhou, China). The mRNA with PolyA was specifically captured by two rounds of purification using oligo (dT) magnetic beads (25-61005, Thermo Fisher Scientific, United States). The captured mRNA was fragmented under high temperature conditions and treated at 94°C for 5–7 min. The fragmented RNA was used to synthesize cDNA. Then, *Escherichia coli* DNA polymerase I [New England Biolabs, Ipswich, MA, United States (NEB)] and RNase H (NEB) were used for double strand synthesis. These complex double strands of DNA and RNA were transformed into DNA double strand, dUTP solution (Thermo Fisher Scientific, United States) was incorporated into the double strand, and the ends of the double stranded DNA were complemented to the flat ends. An A-base was then added to each end to enable it to ligate with a connector with a T-base at the end, and the fragment size was then screened and purified using magnetic beads. The second strand was digested with UDG enzyme (NEB), and then the library with a fragment size of 300 ± 50 bp was formed by PCR—pre-denaturation held at 95°C for 3 min, denaturation at 98°C for a total of 8 cycles of 15 s each, annealing to 60°C held for 15 s, extension at 72°C for 30 s, and final extension held at 72°C for 5 min. Finally, it was double-end sequenced using illumina Novaseq^TM^ 6000 (LC Bio Technology CO., Ltd. Hangzhou, China) in PE150 sequencing mode according to standard practice. The original sequencing reads have been submitted to the SRA at NCBI (Accession number: PRJNA741714).

#### Quality Assessment of Sequencing Results

Low quality and duplicate sequences were removed from the raw reads to obtain clean data, and then the clean reads were compared with the fourth version of the B73 maize reference genome (version ZmB73_5a.59)^1^ using the HISAT2 (Hisat2-2.0.4) software (Kim et al., 2015). Finally, RSEM software was used to detect the expression level of the genes (Li and Dewey, 2011).

#### Analysis of Differentially Expressed Genes

Genes were analyzed for significant differences between the comparison groups using the R package DESeq2 (Pertea et al., 2015), and genes with differential fold FC (Fold change) > 2-fold or FC < 0.5 and *p*-value < 0.05 were defined as DEGs for screening. The screened DEGs were analyzed for GO (Gene Ontology) and KEGG (Kyoto Encyclopedia of Genes and Genomes) enrichment.

#### Weighted Gene Co-expression Network Analysis

A gene co-expression network was constructed from the expression data of 48 samples of genes after deep sowing stress and exogenous GA_3_, BR and SL treatment using R-package WGCNA (Langfelder and Horvath, 2008). The threshold values were set as follows: the mean gene expression FPKM value was 1, the higher the similarity threshold fold to control module fusion, the lower the similarity required for fusion of the 2 modules, the fold value was set to 0.5, and the minimum number of genes within the module was set to 30. A gene expression adjacency matrix was constructed and used to analyze the network topology. In addition, module correlation analysis was performed by module eigenvalues and phenotypic trait data, and Pearson correlation was used to calculate the correlation coefficients between phenotypic trait data and gene module eigenvalues, then their correlation heat maps were drawn. The OmicShare tool^2^ was used to map the network visualization of genes within the module.

#### qRT-PCR of Differentially Expressed Genes

For the accuracy and reliability of the experimental results, we used total RNA from each treatment material for library construction. The cDNA was reverse transcribed with RNA simple total RNA Kit (Tiangen, Shanghai, China). Ten DEGs were randomly selected from two inbred lines under deep sowing stress, and their specific primers (Supplementary Table 1) were designed by Primer-BLAST on NCBI. qPCR amplification was performed on quantum Studio 5 real-time PCR system (Thermo Fisher Scientific, MA, United States) using super real premix plus (SYBR Green) (Tiangen, Shanghai, China). Each treatment had three biological replicates. In addition, each real time PCR was performed at 20 μL. The reaction volume included 10 μL SuperReal PreMix Plus, 6 μL ddH_2_O, 0.8 μL forward primer (10 μmol/L), 0.8 μL reverse primer, 0.4 μL Rox reference dye and 2 μL template cDNA. The amplification procedure was described with reference to Chen et al. (2020). Actin gene was used as the internal reference gene (Zeng et al., 2019), and the gene expression was analyzed by 2^–ΔΔCT^ calculation method.

#### Statistical Analysis of Data

The above experimental data were statistically plotted by Microsoft Excel 2019, and the one-way ANOVA (*P* < 0.05) was analyzed by IBM SPSS 24.0 software.

## Results

### Morphological and Hormone Analysis of Maize Inbred Lines Treated With Exogenous Substances

**FIGURE 1:**
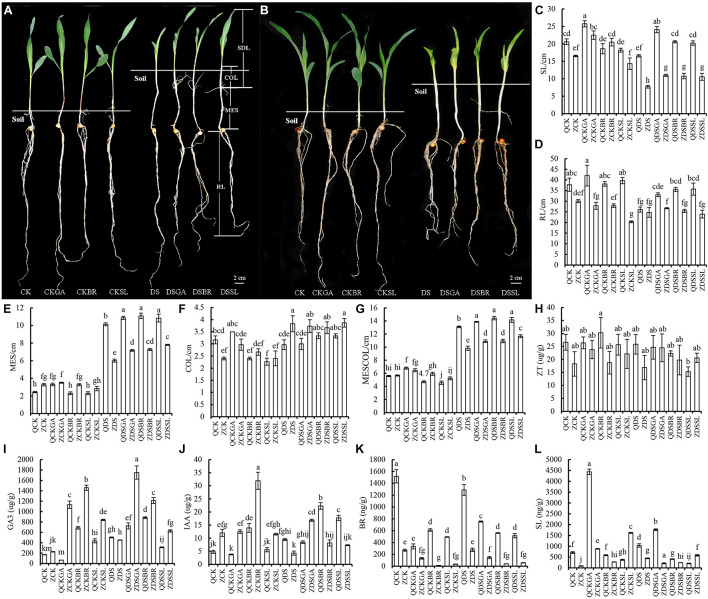
Changes of morphology and endogenous hormone levels of maize inbred lines treated with exogenous hormones. Morphology of **(A)** Qi319 and **(B)** Zi330 under different treatments. **(C–L)** Phenotypic indexes and endogenous hormones contents in the mesocotyl under different treatments. CK, normal sowing; CKGA, after treatment with exogenous GA_3_ under normal sowing; CKBR, after treatment with exogenous BR under normal sowing; CKSL, after treatment with exogenous SL under normal sowing; DS, deep sowing stress; DSGA, after treatment with exogenous GA_3_ under deep sowing; DSBR, after treatment with exogenous BR under deep sowing; DSSL, after treatment with exogenous SL under deep sowing; Q, Qi319; Z, Zi330; MES, Mesocotyl length; COL, coleoptile length; MESCOL, the sum of mesocotyl and coleoptile; SDL, seedling length; RL, root length.

Deep sowing tolerant and deep sowing sensitive lines (Qi319 and Zi330, respectively) were exposed to a range of concentrations of BR, GA_3_, and SL at normal and deep sowing depths. Compared with normal sowing depth, the MES, COL, and MESCOL of sensitive inbred line Zi330 increased significantly under deep sowing stress (*P* < 0.05), and the MES, MESCOL, and SDL of Qi319 increased significantly under deep sowing stress, but the changes of MES and MESCOL in Zi330 were significantly less than those in Qi319. In addition, compared with deep sowing stress, after applying exogenous GA_3_, BR, and SL under deep sowing stress, the MES, MESCOL, and SDL of Zi330 increased significantly, and the MES, MESCOL, and SDL of Qi319 increased significantly, while the degree of change from 330 is significantly greater than Qi319 (Figures 1A–G). The above results showed that exogenous hormone had significant alleviation effect on two maize inbred lines under deep sowing stress, and the mitigation effect of deep sowing sensitive inbred line Zi330 was more obvious.

In order to further understand the physiological response of maize inbred lines to different exogenous substances, we measured the changes of endogenous hormones (ZT, GA_3_, IAA, BR, and SL) in maize mesocotyl under different treatments (Figures 1H–L). There was no significant change in ZT levels in either line under any of the treatments (Figure 1H). Therefore, we speculated that ZT was not a major regulatory hormone in the top soil emergence of maize seedlings. Compared with deep sowing stress, the contents of endogenous GA_3_ and IAA in the two lines were significantly increased, while the content of endogenous hormones BR was significantly decreased after treatment with exogenous GA_3_, BR and SL under deep sowing stress (*P* < 0.05). The changes of endogenous SL were different in the two inbred lines. When exogenous BR was added, the content of endogenous SL decreased in both inbred lines. However, the content of endogenous SL increased after adding GA_3_, and decreased after adding exogenous SL in Qi319. The changes in Zi330 were just opposite to that in Q319. Therefore, we speculate that after adding exogenous GA_3_, BR, and SL, these lines respond to deep sowing stress by increasing endogenous GA_3_ and IAA contents. The decrease of endogenous BR after adding exogenous GA_3_ may be due to the lower optimal level of them in deep sowing. When exogenous BR and SL were applied, the maize didn’t need to produce as much endogenous BR in order to counter the negative effects of deep sowing.

### Cytological Observation of Maize Mesocotyl

**FIGURE 2:**
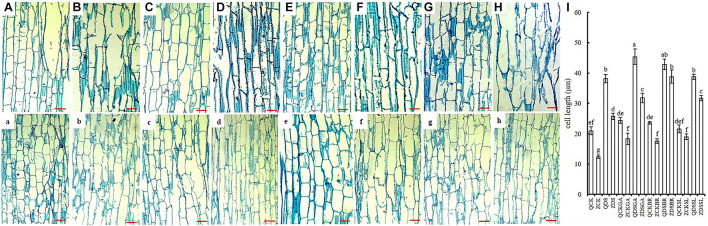
The longitudinal structure of mesocotyl in different maize inbred lines under different treatments. The top row shows Qi319 in treatment groups **(A)** CK, **(B)** DS, **(C)** CKGA, **(D)** DSGA, **(E)** CKBR, **(F)** DSBR, **(G)** CKSL, **(H)** DSSL, and **(I)** cell length in two inbred lines under each treatment. **(a–h)** The bottom row shows the same order of treatment groups as A-H for Zi330. All bars are 12 μm.

The middle position of maize mesocotyl under different sowing depth and different exogenous hormone was cut longitudinally. The cell length of Qi319 was significantly longer than Zi330 under normal sowing depth (Figures 2A,a). When the seeds were sown at 15 cm depth, the cells of Qi319 elongated significantly, and even some of them ruptured due to excessive elongation (Figure 2B). Cells from Zi330 also elongated. However, compared with Qi319, Zi330 had less elongation (Figure 2b). We speculate that this may be one of the reasons for the strong resistance of Qi319 under deep sowing. When exogenous GA_3_, BR, and SL were added under normal sowing depth, and the cell elongation was not obvious (Figures 2A,C,E,G,a,c,e,g), while cell elongation was more obvious and maintained cell integrity under deep sowing (Figures 2B,D,F,H,b,d,f,h). Compared with Qi319, cell elongation of Zi330 was more obvious after adding exogenous GA_3_, BR and SL under deep sowing condition (Figure 2I).

### RNA Quality Control and Library Construction

Maize inbred lines Qi319 and Zi330 were used as materials to obtain transcriptome information under deep sowing stress and alleviated by exogenous hormones. Mesocotyls were collected under normal treatment, deep sowing stress, and addition of exogenous GA_3_, BR and SL under normal and deep sowing stress. To ensure the reliability and comprehensiveness of sequencing data quality, before constructing the library, we used Agilent 2000 biological analyzer to detect the total RNA concentration and quality of the extracted mesocotyl samples. The RNA integrity and purity of 48 samples were good, reaching the standard of RNA sequencing (Supplementary Table 2). Using Illumina NovaSeq^TM^ 6000, the 48 libraries were sequenced and the sequencing mode was PE150. An average of 32754154-5584562664 reads were obtained for each sample. After removing the sequencing connector and low-quality sequencing data, each sample obtained 41958844 valid data on average, with Q30% value ranging from 97.37 to 97.96%, and GC content ranging from 50.50 to 53.50%. Using maize genome V4 as reference genome, the average number of reads per sample was 35614689, and the comparison rate was 87.88% (Supplementary Table 3).

### Differentially Expressed Gene Analysis

**FIGURE 3:**
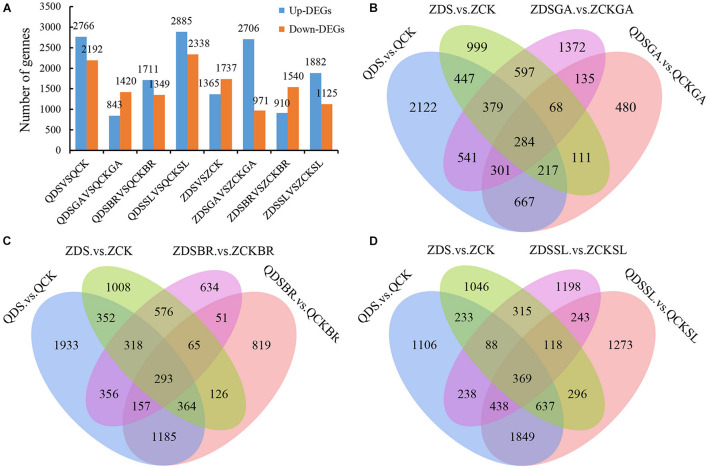
Analysis of DEGs under different comparison groups. **(A)** The distribution of the number of DEGs in different comparison groups; **(B–D)** Venn diagram analysis of normal sowing depth, deep sowing stress and adding exogenous GA_3_, BR, and SL and normal sowing depth and deep sowing stress, respectively.

We next sought to identify genes potentially involved in deep sowing tolerance using RNA-Seq (see section “Materials and Methods”) to compare gene expression in each sample group. Differentially expressed genes (DEGs) were here defined as genes with a fold-change >2 or <0.5 and with a *p*-value < 0.05. The numbers of DEGs in the two lines under deep sowing stress and exogenous hormone treatment, as well as the common and unique distribution of genes in each comparison group were shown (Figure 3). In Qi319, 2,766 DEGs were up regulated and 2,192 were down regulated in the deep-sown group. In Zi330, 1,365 DEGs were up regulated and 1,737 were down regulated, indicating that the deep sowing tolerant line Qi319 enhanced its deep sowing tolerance through more up regulated or down regulated DEGs (Figure 3A). Compared with GA_3_ under normal sowing depth, 843 DEGs were up regulated and 1,420 DEGs were down regulated in Qi319 with GA_3_ under deep sowing stress. However, 2,706 DEGs were up regulated and 971 DEGs were down regulated in Zi330 after adding exogenous GA_3_ under deep sowing stress, which indicated that deep sowing sensitive inbred lines could also enhance their deep sowing tolerance through more up regulated DEGs under exogenous GA_3_ treatment. Compared with BR under normal sowing depth, 1,711 DEGs were up regulated and 1,349 DEGs were down regulated in Qi319 with BR under deep sowing stress. However, 910 DEGs were up regulated and 1,540 DEGs were down regulated in Zi330 after adding exogenous BR under deep sowing stress. Compared with SL under normal sowing depth, 2,885 DEGs were up regulated and 2,338 DEGs were down regulated in Qi319 with SL under deep sowing stress. However, 1,882 DEGs were up regulated and 1,125 DEGs were down regulated in Zi330 after adding exogenous SL under deep sowing stress. This indicated that the deep sowing resistant inbred line was more sensitive to exogenous BR and SL than the deep sowing sensitive inbred line under deep sowing stress. Similarly, the analysis of the distribution of common and unique DEGs in Venn diagram fully illustrated that application of the hormones was mitigating the negative effects of deep sowing (Figures 3B–D).

### Functional Analysis of Differentially Expressed Genes

In order to further understand the biological function of DEGs in the above comparison groups, we carried out GO enrichment analysis. GO terms include categories of biological processes, cellular components and/or molecular functions for a given gene product. Under deep sowing stress, DEGs in Qi319 were most enriched in the biological processes of oxidation-reduction, phosphorylation and regulation of transcription, DNA-templated. Cellular components in which DEGs were most enriched were the membrane, followed by integral component of membrane and nucleus. The most enriched molecular functions were ATP binding, transferase activity, metal ion binding, hydrolase activity and oxidoreductase activity. We identified a gibberellin receptor GID1 involved in the hydrolase activity, and two auxin-responsive proteins and an Aux/IAA-transcription factor 4 involved in regulation of transcription, DNA-templated. The results of significant enrichment of DEGs of Zi330 in biological processes, cell component and molecular functions were basically consistent with those of Qi319, but Qi319 had more DEGs in the same enrichment term. Therefore, we speculated that oxidation-reduction process, transcriptional regulation and energy transport were involved in the regulation mechanism of deep sowing tolerance. Overall, the same biological processes, cellular compartments, and molecular functions described above were also enriched in deep sowing plants treated with hormones, with the difference being that there were more DEGs belonging to each enriched term after adding exogenous GA_3_ and SL under deep sowing stress. This indicated that exogenous GA_3_ and SL enhanced maize deep sowing tolerance by up or down regulated more DEGs. After adding exogenous BR under deep sowing stress, the two lines had almost the same number of DEGs in each enriched term, which indicated that the effect of BR application was minimal. Additionally, plants in all three hormone treatment groups showed enrichment in lignin metabolism, protein kinase activity, and cell wall organization. Therefore, we speculated that they may play an important role in maize deep sowing tolerance and exogenous hormones alleviating deep seeding stress (Supplementary Figures 1–4).

**FIGURE 4:**
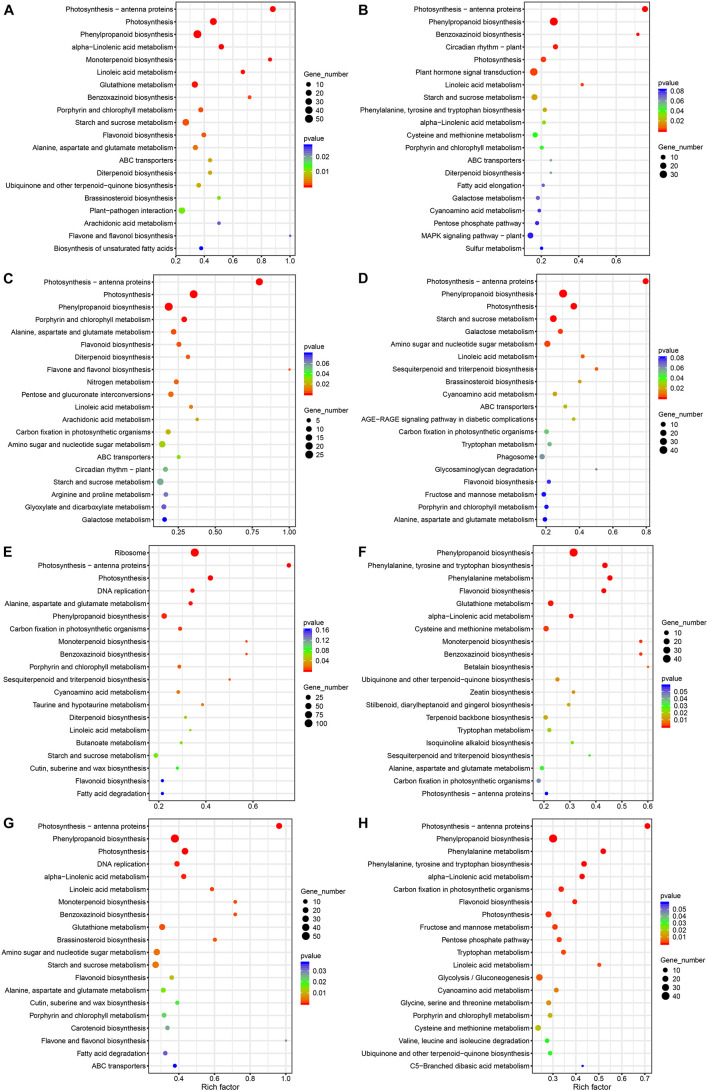
KEGG analysis of DEGs in different comparison groups. **(A)** QDS vs. QCK; **(B)** ZDS vs. ZCK; **(C)** QDSGA vs. QCKGA; **(D)** QDSGA vs. ZDGA; **(E)** QDSBR vs. QCKBR; **(F)** ZDSR vs. ZDBR; **(G)** QDSSL vs. QCKSL; **(H)** ZDSSL vs. ZDSL.

In order to further understand the functional differences of DEG enrichment under deep sowing stress and with exogenous GA_3_, BR, and SL treatment, we analyzed the metabolic pathway annotations of the DEGs in these comparison groups (Figure 4 and Supplementary Table 4). We observed that the number of DEGs annotated in metabolic pathways differed between comparison groups, ranging from 293 to 625; the number of metabolic pathways significantly enriched was also different, ranging from 90 to 113. The number of significantly enriched pathways was also different between the two groups. For example, ZDSGA vs. ZCKGA showed the least enrichment in number of metabolic pathways in our comparison groups (12), while QDS vs. QCK showed enrichment in the highest number of pathways in our comparison groups (25), indicating that there were differences in pathway metabolism between different treatments. Compared with the control group, the enriched pathways in Qi319 under deep sowing stress included phenylpropanoid biosynthesis, photosynthesis, starch and sucrose metabolism, plant-pathogen interaction and brassinosteroid biosynthesis, but brassinosteroid biosynthesis and plant-pathogen interaction were not significantly enriched in Zi330, indicating that these pathways may play an important role in maize deep sowing stress (Figures 4A,B). In addition, plant hormone signal transduction was significantly enriched in Zi330 under deep sowing stress, in which we identified two auxin-responsive proteins and an Aux/IAA-transcription factor 4. Under GA_3_ treatment, the enriched pathways in QDSGA vs. QCKGA included phenylpropane biosynthesis and photosynthesis-antenna proteins; metabolic pathways enriched in Zi330 included those enriched in Qi319, with additional enrichment in sesquiterpenoid and triterpenoid biosynthesis, starch and sucrose metabolism, and brassinosteroid biosynthesis. These unique metabolic pathways may be related to the significant elongation of mesocotyl under deep sowing stress (Figures 4C,D). Under BR treatment, there was little overlap in metabolic pathway enrichment between the two lines, with Qi319 showing enrichment in ribosome, phenylpropane biosynthesis, photosynthesis and photosynthesis-antenna proteins pathways, and Zi330 showing enrichment in phenylpropane biosynthesis, glutathione metabolism, tyrosine and tryptophan biosynthesis and phenylalanine metabolism pathways. Under SL treatment, phenylpropane biosynthesis, photosynthesis and starch and sucrose metabolism were the main metabolic pathways enriched in Qi319; however, phenylpropanoid biosynthesis, photosynthesis, glycolysis/gluconeogenesis and photosynthesis antenna proteins were enriched in Zi330, which indicated that treatment with exogenous SL mainly altered expression of DEGs related to phenylpropanoid biosynthesis, photosynthesis and sugar metabolism pathway in maize mesocotyl elongation under deep sowing stress (Figures 4G,H).

### Gene Co-expression Network Analysis

**FIGURE 5:**
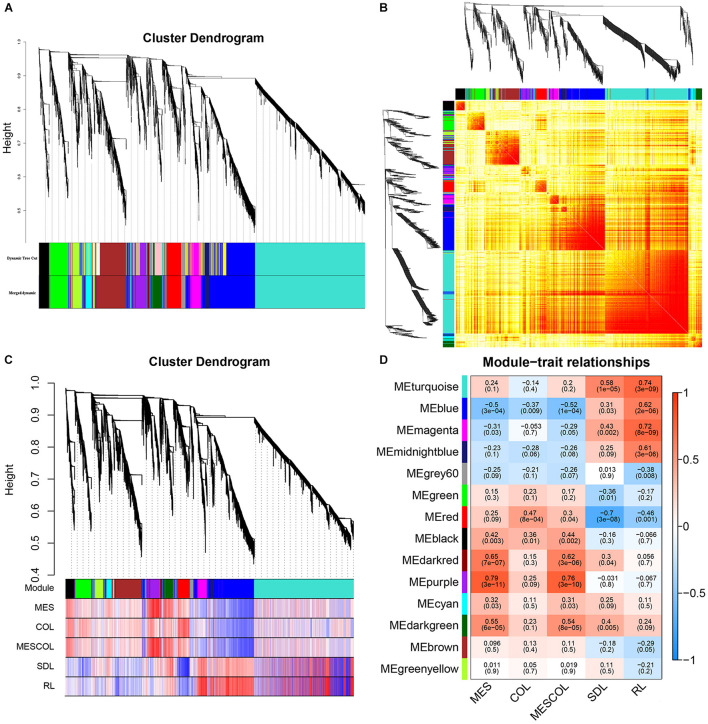
Gene cluster analysis and correlation analysis between phenotype and module. **(A)** Hierarchical clustering analysis of co-expression genes. **(B)** Correlated heat maps between modules. **(C)** Correlation between gene module and phenotype. **(D)** Heat map of correlation between gene module and phenotype.

In order to identify co-expression patterns among DEGs, we used WGCNA, which clusters gene sets with similar expression patterns were into modules. Fourteen co-expression modules were constructed from the expression data of 48 samples with FPKM values >1 (Figures 5A,B). We then explored the relationship between modules and specific traits/phenotypes of our plants under normal sowing depth, deep sowing stress and exogenous hormones treatment under normal and deep sowing. Deep sowing tolerance in maize had previously been shown to primarily involve functions related to mesocotyl elongation. Therefore, our interest was focused on analyzing modules with which mesocotyl elongation was significantly positively correlated with the purple and darkred modules (Figures 5C,D).

### Functional Analysis of Genes in Correlated Modules

**FIGURE 6:**
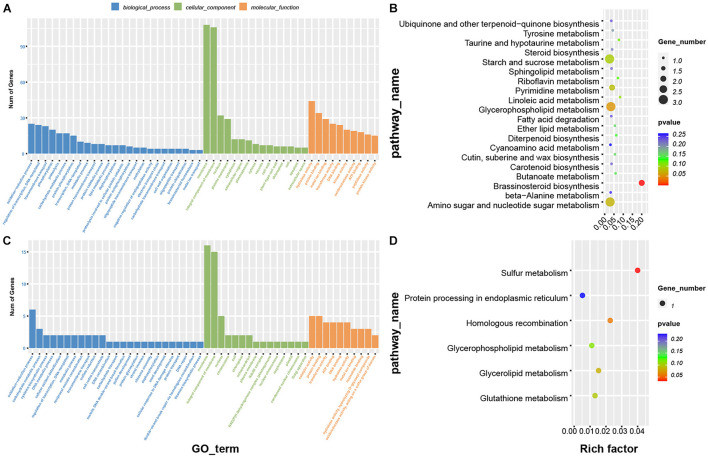
Functional analysis of genes in the phenotypic significant enrichment module. Panels **(A,B)** represent GO and KEGG enrichment analysis of genes in purple module, **(C,D)** represent GO and KEGG enrichment analysis of genes in dark red module.

To further understand the biological functions of the genes in these modules, we performed GO and KEGG enrichment analysis (Figure 6) as discussed above. GO biological processes enriched in the purple module were oxidation-reduction process, transcriptional regulation, DNA-templated and transmembrane transport; enriched molecular functions were hydrolase activity, protein binding, metal ion binding, and protein kinase activity (Figure 6A). The biological processes enriched in the darkred module were oxidation-reduction process, carbohydrate metabolic process and cysteine biosynthetic process; enriched molecular functions were catalytic activity and transferase activity (Figure 6C). KEGG enrichment analysis showed that the genes in the purple module were most commonly involved in starch and sucrose metabolism, amino sugar and nucleotide sugar metabolism, and brassinosteroid biosynthesis pathways (Figure 6B). Enriched KEGG functions in the darkred module were most commonly involved in sulfur metabolism, homologous recombination and glycerolipid metabolism (Figure 6D). We therefore speculated that these pathways may have an important role in deep sowing tolerance.

### Analysis of Hub Genes Interaction Network in the Module

**FIGURE 7:**
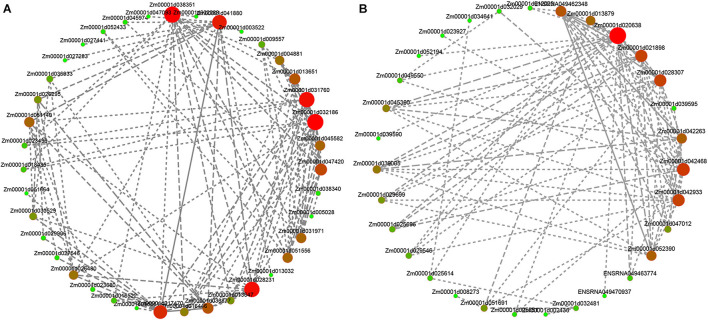
Analysis of hub genes network interaction in phenotypic significant enrichment module. **(A)** Network interaction analysis of hub genes in purple module. **(B)** Network interaction analysis of hub genes in crimson module. The size and color gradient of the dots represent the high or low soft threshold of connectivity, with the redder color of the dots representing a higher soft threshold of connectivity.

To determine specific genes most likely to be important in deep sowing tolerance, we additionally used the expression data to generate a co-expression network. The network was used to identify “hub genes” for treatment groups. This term referred to genes with high connectivity in a gene interaction network. In this study, the five genes with the highest kME values (Eigengene connectivity) in each of the purple and the dark red modules were classified as hub genes. The hub genes and their interacting genes were used to map the gene co-expression network (Figure 7 and Supplementary Table 5). Within the purple module, the hub genes were adenine nucleotide alpha hydrolases-like superfamily protein and beta-galactosidase 9 involved in hydrolase activity, clathrin assembly protein involved in clathrin binding, ribonuclease 1 involved in transcriptional regulatory process, and Sec14p-like phosphatidylinositol transfer family protein was not annotated to any biological process. Within the darkred module, the hub genes were probable inactive poly (ADP-ribose) polymerase SRO1 involved in NAD + ADP-ribosyltransferase activity, protein GAMETE EXPRESSED 1 involved in components of the membrane, zinc finger protein ZAT11 and recombination protein51 gene a involved in transcriptional regulation, structural maintenance of chromosomes protein 2-2 involved in protein binding. Therefore, we speculated that transcriptional regulation, hydrolase activity, protein binding, and plasma membrane played important roles in deep sowing tolerance in maize and in the alleviating deep sowing stress by the exogenous hormones GA_3_, BR, and SL. In addition, previous studies believed that auxin was essential for the elongation of maize mesocotyl (Zhao and Wang, 2010; Sharova et al., 2012). In the purple module, we found that auxin-responsive proteins IAA33 and IAA26 (Zm00001d026480, Zm00001d039513) and Aux/IAA-transcription factor 7 (Zm00001d045026) were involved in auxin-activated signaling pathway.

### qRT-PCR Validation

**FIGURE 8:**
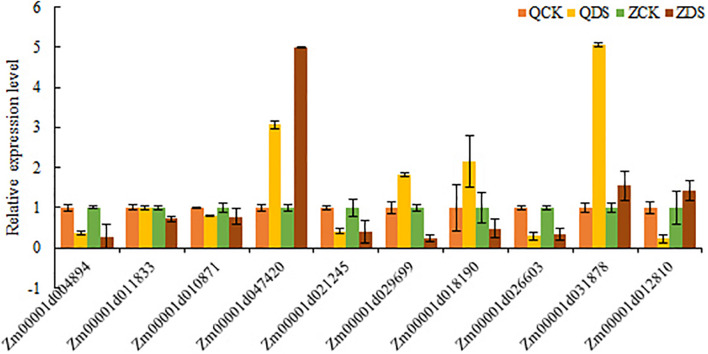
qRT-PCR verification of RNA-seq sequencing results.

To verify the reliability of the sequencing results of transcripts, we randomly selected 10 common DEGs under deep sowing stress treatment in two inbred lines for qRT-PCR analysis (Figure 8). The relative expressions of Zm00001d004894, Zm00001d01183, Zm00001d010871, Zm00001d047420, Zm00001d021245, Zm00001d029699, Zm00001d026603, and Zm00001d031878 were consistent with that of the RNA-seq results of two inbred lines under deep sowing stress, while the relative expression of Zm000011d018190 was <1, which was contrary to that of Zi330 under deep sowing stress. In addition, Zm00001d012810 was up regulated in transcriptional sequencing and down regulated in qRT-PCR. Based on the above analysis, the expression trends of 8 out of 10 genes were consistent with the sequencing results, which fully showed that our sequencing results are reliable.

## Discussion

### Phenotypic Analysis of Exogenous Substances to Alleviate Deep Sowing Stress

When crop seeds are sown at a deeper depth than normal sowing, soil resistance increases during seedling emergence due to the scarcity of air in the deep soil, which can cause different degrees of damage to the plant. Studies have shown that deeper sowing significantly reduces seed vigor, prolongs the time for seedlings to emerge from the soil, increases nutrient depletion and negatively affects seedling growth and development (Sanusan et al., 2009; Peng et al., 2016). In recent years, research into maize deep sowing tolerance mainly focused on the effect of deep sowing stress on the phenotype of maize seedlings. Within a certain range, increasing sowing depth had been shown in maize to be correlated with decreasing emergence rate, root number, root-shoot ratio, and seedling integrity, and with increasing emergence time, SDL, MES, COL, RL, SDW, and MESW (Peng et al., 2014, 2016; Liu et al., 2017). Consistent with these prior findings, our results showed that the length of MES, MESCOL, and RL in both Zi330 and Qi319 were significantly increased; however, in contrast, we found that SDL was significantly shortened. In addition, compared with the deep sowing stress, MES, MESCOL, and SDL in both lines were significantly increased by exposure to exogenous GA_3_, BR, and SL under deep sowing stress. This indicated that the treatment of maize under deep sowing stress by exogenous GA_3_, BR, and SL could effectively alleviate the negative effects of deep sowing stress on maize.

### Analysis of Relative Hormone Content in Maize Mesocotyl Under Exogenous Hormone Treatment

Previous studies had shown that the elongation of maize mesocotyl under deep sowing was largely regulated by the action of various endogenous plant hormones such as IAA, GA_3_, ZT, ABA, ETH, BR, and SL (Du et al., 2009; Hu et al., 2014; Kutschera and Wang, 2016). Simultaneous application of GA_3_ and gibberellin synthesis inhibitor uniconazole (UCZ) under deep sowing had shown that GA_3_ and IAA levels in maize mesocotyl were increased (Zhao G. W. et al., 2009b; Sharova et al., 2012). Our results were consistent, indicating that exogenous application of GA_3_ alleviated deep sowing stress by increasing IAA content and thus promoting mesocotyl elongation. IAA was a master control factor of maize mesocotyl elongation, acidifying and relaxing the epidermal cell wall of maize mesocotyl and thus achieving cell elongation (Kutschera and Wang, 2016). BR and IAA were involved in regulating the development of maize mesocotyl elongation by the same mechanism, and they interacted synergistically to directly share the promoter of HBI1, a transcription factor that regulated cell wall relaxation, to synergistically regulate gene expression and mesocotyl elongation (Deng et al., 2012; Hartwig et al., 2012; Fan et al., 2014). In the present study, endogenous IAA increased after the addition of exogenous GA_3_, BR, and SL, while endogenous BR decreased, likely because BR acted synergistically with IAA, and the increase in IAA content inhibited the synthesis of BR and thus led to its decrease. In rice, CTK promoted mesocotyl elongation by promoting cell division, while SL can inhibit the division of rice mesocotyl cells and thus negatively affected the elongation of the mesocotyl (Hu et al., 2010, 2014). In this study, the ZT content did not change significantly and the SL content decreased after the application of exogenous GA_3_, BR, and SL under deep sowing stress, indicating that exogenous GA_3_, BR, and SL promoted the elongation of the mesocotyl under deep sowing stress, mainly by promoting the elongation or expansion of the cell volume as opposed to cell division.

### Cytological Analysis of Maize Mesocotyl Under Exogenous Hormone Treatment

Cytologically, the elongation of mesocotyl is a result of increasing cell numbers and cell volume. Zhao G. W. et al. (2009b) showed that elongation of maize mesocotyl under deep sowing stress was primarily due to cell elongation rather than increasing cell numbers. This was consistent with the results of the present study. They also showed that both exogenous GA_3_ and IAA could alleviate deep sowing stress and elongate maize mesocotyl cells to achieve topsoil emergence; however, they also found that maize mesocotyl cells treated with GA synthesis inhibitor were not only significantly shorter in length but also significantly reduced in cell number, indicating that GA synthesis inhibitor inhibited the elongation of mesocotyl cells and cell division. Thus, it was hypothesized that the application of exogenous GA_3_ caused complex physiological functions. In this study, the mitigation effects of three exogenous hormones, GA_3_, BR, and SL, were found to vary depending on whether they were applied to a deep sowing tolerant or sensitive line. This highlighted the importance of testing the effects of exogenous hormones on specific lines prior to using such treatments in the field. In addition, Li et al. (2012) suggested that the mesocotyl cells of weedy and cultivated rice were not of the same length in the upper, middle and lower zones. To investigate the morphological variation of longitudinal cells in rice mesocotyl, Wang (2017) showed that the number of mesocotyl cells, as well as the cell size, differed between sections when the mesocotyl was divided into three parts evenly from top to bottom for longitudinal cutting. In this study, only the middle mesocotyl was used to observe its microstructure, and it was found that the mesocotyl cells changed differently in different inbred lines under deep sowing conditions. Our results are of some reference significance; however, to observe the cytology of the entire mesocotyl of maize under deep sowing conditions, it is also necessary to subdivide the mesocotyl into multiple parts for a comprehensive study.

### Analysis of Differentially Expressed Genes Under Deep Sowing Stress and Alleviated by Exogenous Substances

So far, there has been very limited progress in molecular theoretical studies on the mechanisms of deep sowing stress, GA_3_, BR, and SL on regulation of mesocotyl elongation. We found that GA_3_, BR, and SL alleviated deep sowing stress, and the DEGs in the two inbred lines were mainly found in the functions related to oxidation-reduction processes, plasma membrane and cell wall organization; these genes mainly included cellulose synthase, expansin and glucanase, and oxidase. Fagard et al. (2000) found that cellulose synthase was necessary for normal cell elongation. Expansin was also a key regulator of cell and cell wall elongation (Cosgrove, 2000). In this study, we found that expansin was up-regulated in the two inbred lines under the stress of deep seeding and alleviated by exogenous GA_3_, BR, and SL. In addition, glucanase may play an important role in cellulose synthesis and cell elongation (Sato et al., 2001; Chen et al., 2003). It was reported that exogenous GA_3_ was demonstrated to increase glucanase activity in maize and barley (*Hordeum vulgare* L.) (Carpita and Kanabus, 1988; Kotake et al., 2000). Interestingly, the glucanases identified in Zi330 were up regulated under deep sowing stress and after GA_3_ treatment under deep sowing, whereas four glucanases (Endoglucanase 2, Endo-1%2C4-beta-glucanase Cel1, Endoglucanase 25, and beta-glucanase 3) were down regulated in Qi319 under sowing stress (Supplementary Table 6). We hypothesized that the proportion of glucan in the cell wall of deep-seeded tolerant maize mesocotyl cells had reached a metabolic equilibrium point, and the increased glucanase activity of the deep sowing sensitive inbred line reduced the proportion of glucan in the cell wall and improved favorable conditions for mesocotyl elongation.

Lignin is an important component of the secondary wall of plant cells and is directly related to the amount of cell wall elasticity. It is believed that cell wall relaxation decreased with increasing lignin content, and when lignin content was too high, it caused inhibition of elongation growth of cells (Zhou, 2005). During cell wall lignification, the accumulation of H_2_O_2_ induced peroxidase (POD) to oxidize cell wall monolignans to free radicals and polymerize to produce lignin, which leaded to cell wall sclerosis and hindered elongation growth (Karpinska et al., 2001). In this process, the sources of H_2_O_2_ primarily included the three pathways of reduced nicotinamide adenine dinucleotide phosphate oxidase (NADPH), POD, and polyamine oxidase (PAO) (Frahry and Schopfer, 2001; Jiang and Zhang, 2002; Yoda et al., 2003). Among the genes involved in the lignin biosynthesis process after deep sowing stress, a possible MYB-transcription factor 92 was up regulated in Zi330 but down regulated in Qi319, while the rest of the DEGs were down regulated in both inbred lines but were more highly expressed in Qi319 (Supplementary Table 7). After GA_3_ treatment, the DEGs involved in the lignin biosynthesis process were down regulated in both inbred lines, while Qi319 had a higher number of genes (dominated by cytochrome P450 and cinnamyl alcohol dehydrogenase). Most DEGs involved in the lignin biosynthesis process were down regulated in both inbred lines after BR treatment, but Zi330 had a higher number of genes (dominated by cinnamyl alcohol-related genes). The DEGs involved in the lignin synthesis under exogenous SL treatment were down regulated in Qi319, except for the probable cinnamyl alcohol dehydrogenase 6 (Zm00001d020400). Interestingly, all genes related to lignin biosynthesis were up regulated in Zi330, suggesting that Zi330 may require lignin to provide rigid support for the mesocotyl of seedlings during topsoil emergence under exogenous SL treatment. DEGs involved in redox processes, such as peroxidase 16 (Zm00001d014608) and peroxidase 64 (Zm00001d027411), were up regulated in both inbred lines after exogenous GA_3_, BR, and SL treatment. This suggested that peroxidase may be induced by exogenous GA_3_, BR, and SL, thereby scavenging the accumulation of H_2_O_2_ to reduce the oxidative polymerization of cell wall monolignans into lignin to harden the cell wall and reduce the degree of blocked cell elongation. These genes may be responsible for cell wall synthesis and cell elongation, suggesting that they may play an important role in the elongation of mesocotyl under deep sowing stress in Qi319 and Zi330 due to GA_3_, BR and SL induction.

Endochitinase played an important role in the defense against fungal pathogens containing chitin (Navarro-González et al., 2019). Zhao G. et al. (2009a) first suggested that endochitinase can be induced by GA_3_*o* and invlved in deep sowing tolerance and mesocotyl elongation in maize. Under deep sowing stress, six basic endochitinase genes related to the amino- and nucleotide sugarm etabolism pathways were down regulated in Zi330; among them, endochitinase precursor 4 (Zm00001d017152), basic chitinase A (Zm00001d009936) and acidic chitinase (Zm00001d049727) were up regulated in Qi319 (Supplementary Table 8). After GA_3_ treatment, the four endochitinases were up regulated in Qi319, while the two basic endochitinases B were down regulated in Zi330. In addition, under exogenous BR treatment, we found that both basic endonuclease A and B (Zm00001d009936, Zm00001d029794) were down regulated in Zi330 (which was consistent with the results under deep sowing stress), and that endonuclease A (Zm00001d003190), acidic endonuclease (Zm00001d018966, Zm00001d048903) and endonuclease precursor 4 (Zm00001d017152) were up regulated in Qi319. Under exogenous SL treatment, endonuclease A (Zm00001d003190) and three acidic endonucleases were up regulated in Zi330, while basic endonuclease B (Zm00001d027525, Zm00001d027524) remained down regulated. Based on the above analysis, it can be speculated that acidic endonuclease may be a positive factor for mesocotyl elongation under deep sowing stress, whereas basic endonuclease may be a detrimental factor.

Many protein kinase genes were up regulated in the two inbred lines under deep sowing stress and exogenous GA_3_, BR and SL treatment (Supplementary Table 9). The primary difference was that more protein kinase genes were up regulated in Qi319 under deep sowing stress, exogenous BR and exogenous SL treatment, while more protein kinases were up regulated in Zi330 under exogenous GA_3_ treatment. These results indicated that exogenous GA_3_ can improve maize deep sowing tolerance through protein kinase genes. Possible leucine-rich repeat sequence receptor protein kinase family proteins (Zm00001d025002, Zm00001d037672) with protein kinase superfamily proteins (Zm00001d043959) were up regulated in the two inbred lines after exogenous BR and exogenous SL treatment and in Zi330 after exogenous GA_3_ treatment. In addition, many protein kinase genes such as possible LRR receptor-like serine/threonine protein kinase and serine/threonine protein kinase were up regulated in Zi330 under exogenous GA_3_, BR, and SL treatment. Zhao G. et al. (2009a) showed that many possible protein kinases, such as CAMKlike, Raf, LRR, and RLCK, were up regulated in maize under deep sowing stress after exogenous GA_3_ treatment or down regulated after UCZ treatment, which well-illustrated the depth of signal transduction penetration during mesocotyl elongation.

The protein phosphatases 2C constitutes a distinct protein serine/threonine phosphatase family and is involved in the ABA signaling pathway. Under deep sowing stress, six protein phosphatases 2C were down regulated in Zi330, and four protein phosphatases 2C were down regulated in Qi319 (Supplementary Table 10). Under exogenous BR treatment, two probable protein phosphatase 2C (Zm00001d034655, Zm00001d012962) were down regulated in Zi330, and four protein phosphatase 2C (Zm00001d039745, Zm00001d044525, Zm00001d053313, and Zm00001d005071) were down regulated in Qi319. Under exogenous SL treatment, seven and nine protein phosphatase 2C were down regulated in Zi330 and Qi319, respectively. Schweighofer et al. (2004) showed that protein phosphatase 2C was involved in the negative regulation of ABA (a plant growth inhibitory factor) signaling. Several members of the MAPK (mitogen-activated protein kinase) family were found to be directly or indirectly activated by ABA signaling (de Zelicourt et al., 2016). In this study, MAPK20 was found to be down regulated in Qi319 under deep sowing stress after application of exogenous GA_3_, BR, and SL, but up regulated in Zi330 under exogenous GA_3_ treatment. Based on the above analysis, we suggested that exogenous GA_3_, BR, and SL may inhibit the ABA response pathway and thus promote mesocotyl elongation.

It was reported that the transcript abundance of GID1 in *Arabidopsis thaliana* was reduced by exogenous GA_3_ induction (Griffiths et al., 2006). The GA receptor GID1 was down regulated in Qi319 but up regulated in Zi330 after exogenous GA_3_ application. This may be due to the fact that exogenous GA_3_ was saturated to deep sowing resistant inbred lines, but not to deep sowing sensitive inbred lines. After GA binding, GID1 interacted with DELLA protein, which was a repressor of GA signaling, leading to DELLA protein degradation by the proteasome, thus allowing GA signaling (Ueguchi-Tanaka et al., 2007). GID1 mutants in *Arabidopsis thaliana* had been reported to display reduced stem height (Griffiths et al., 2006; Iuchi et al., 2007). Therefore, it was speculated that GID1 may have a role in promoting organ elongation. In addition, we also found that GID1 was up regulated in two inbred lines under the deep sowing of exogenous SL alleviation. We speculated that exogenous SL may promote the combination of GA and GID1, and have a positive effect on the mesocotyl elongation.

At present, the interactions between hormones related to elongation of endosperm cells in maize are still unclear to a large extent. Zhao and Wang (2010) believed that auxin could regulate mesocotyl elongation mainly by increasing IAA synthesis and transportation, and found that increasing IAA concentration could effectively promote maize mesocotyl elongation under deep sowing condition. Sharova et al. (2012) suggested that IAA was the main controlling factor of the mesocotyl elongation in maize. In addition, auxin binding protein was found to be actively involved in promoting the elongation of mesocotyl in maize under deep sowing conditions (Zhao and Wang, 2010). Kutschera and Wang (2016) found that exogenous IAA can significantly promote the elongation of maize coleoptile and mesocotyl *in vitro*, and the mechanism of BR and IAA involved in regulating the growth and development of maize coleoptile and mesocotyl was consistent. These two hormones promoted cell elongation by acidifying and relaxing the epidermal cell wall of maize coleoptile and mesocotyl. Similarly, Pan et al. (2017) showed that exogenous GA_3_ also promoted the elongation of the hypocotyl under deep sowing. In addition, “auxin elongation hypothesis” had been confirmed and extended, and it was reported that auxin binding protein 1 and IAA receptor were involved in auxin/light signal network of maize (Borucka and Fellner, 2012). In this study, we found that two auxin responsive proteins IAA26 and IAA4 (Zm00001d010360, Zm00001d033319) and Aux/IAA transcription factor 4 (Zm00001d033976) were up regulated after applying exogenous GA_3_, BR, and SL under deep sowing stress in Zi330, responsive proteins IAA4 and Aux/IAA transcription factor 4 were up regulated after applying exogenous BR and SL under deep sowing stress in Qi319 by GO and KEGG enrichment. In addition, we identified that the two auxin-responsive proteins IAA33 and IAA26 were up-regulated in two inbred lines under deep sowing stress and exogenous BR alleviating stress. And we also identified that auxin response factor 7 was only up-regulated in deep sowing tolerance inbred line Qi319 under deep sowing stress by WGCNA analysis. Therefore, we speculated that these three genes may be actively involved in the regulation of mesocotyl elongation under deep sowing stress and alleviated by exogenous hormones GA_3_, BR, and SL.

## Conclusion

**FIGURE 9:**
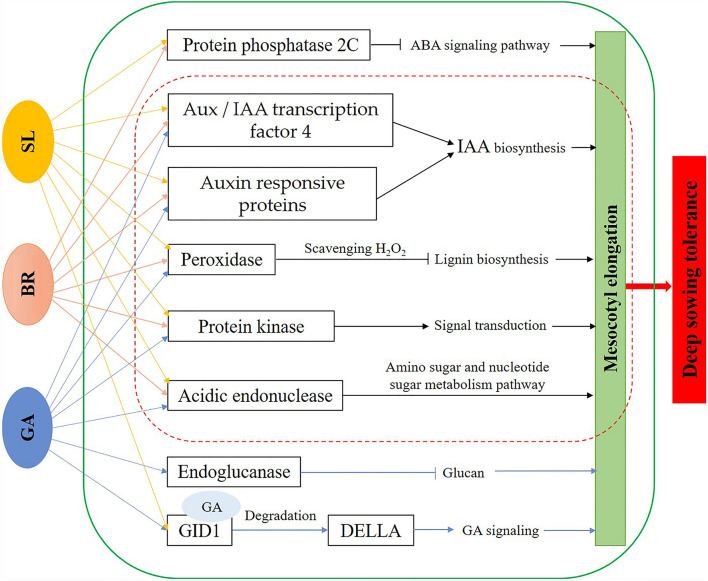
Molecular model of the mechanisms underlying exogenous hormone regulation of deep sowing tolerance in maize seedling mesocotyls. GA, exogenous gibberellins; BR, exogenous brassinosteroids; SL, exogenous strigolactones.

While deep sowing has been proposed as a strategy to deal with drought and water shortage, this method can have a negatives damage of deep sowin impact on the growth and development of plants. We here showed that application of exogenous hormones GA_3_, BR, and SL can effectively alleviate the stress damage of deep sowing in maize. Comparing the phenotypic differences, cytological structure of the mesocotyl, endogenous hormone content, and transcriptional expression profiles between the deep-sowing tolerant inbred line Qi319 and the deep-sowing sensitive inbred line Zi330 under normal sowing, deep sowing stress, and with the addition of different exogenous hormones, we found that maize mitigated damage caused by deep sowing stress through mesocotyl elongation. Based on enrichment of specific functions and metabolic pathways in plants that fared well under deep sowing stress, whether due to genotype or exogenous hormone application, we hypothesized that the mechanism of mesocotyl elongation was caused by increased IAA and GA_3_ content, leading to acidification and relaxation of the mesocotyl epidermal cell wall and therefore to the elongation or expansion of the mesocotyl cells. Transcriptional expression profile analysis showed that genes such as cellulose synthase, expansin and glucanase, oxidase and lignin synthesis may be related to maize deep sowing stress, and genes such as oxidase, endochitinase, protein kinase and 2C protein phosphatase may be related to the alleviation of maize deep sowing stress by exogenous hormones. And, we established the molecular model of the mechanisms underlying exogenous hormone regulation of deep sowing tolerance in maize seedling (Figure 9). Our research not only provides a new perspective to solve the problem of maize planting in drought and water shortage areas, but also provides a reference for the mechanistic research of maize deep sowing tolerance and exogenous hormones alleviating maize deep sowing stress.

## Data Availability Statement

The original contributions presented in the study are publicly available. This data can be found here: National Center for Biotechnology Information (NCBI) BioProject database under accession number PRJNA741714.

## Author Contributions

FC, XJ, MB, and ZZ carried out the transcriptomics analysis and drafted the manuscript. FC, MB, and YP participated in material culture and performed the statistical analysis. FC and YP conceived of the study and participated in its design. XJ, MB, ZZ, and YP helped to draft the manuscript. All authors read and approved the final manuscript.

## Conflict of Interest

The authors declare that the research was conducted in the absence of any commercial or financial relationships that could be construed as a potential conflict of interest.

## Publisher’s Note

All claims expressed in this article are solely those of the authors and do not necessarily represent those of their affiliated organizations, or those of the publisher, the editors and the reviewers. Any product that may be evaluated in this article, or claim that may be made by its manufacturer, is not guaranteed or endorsed by the publisher.

## Footnotes

1
http://ftp.maizesequence.org/

2
www.omicshare.com/tools
