# Supplementary material for: Network Analysis of Different Exogenous Hormones on the Regulation of Deep Sowing Tolerance in Maize Seedlings
Source: Front Plant Sci. 2021 Dec 2;12:739101. doi: 10.3389/fpls.2021.739101 (PMC8674439; doi:10.3389/fpls.2021.739101)
Supplement: Supplementary file 1 [file Data_Sheet_1.DOCX]

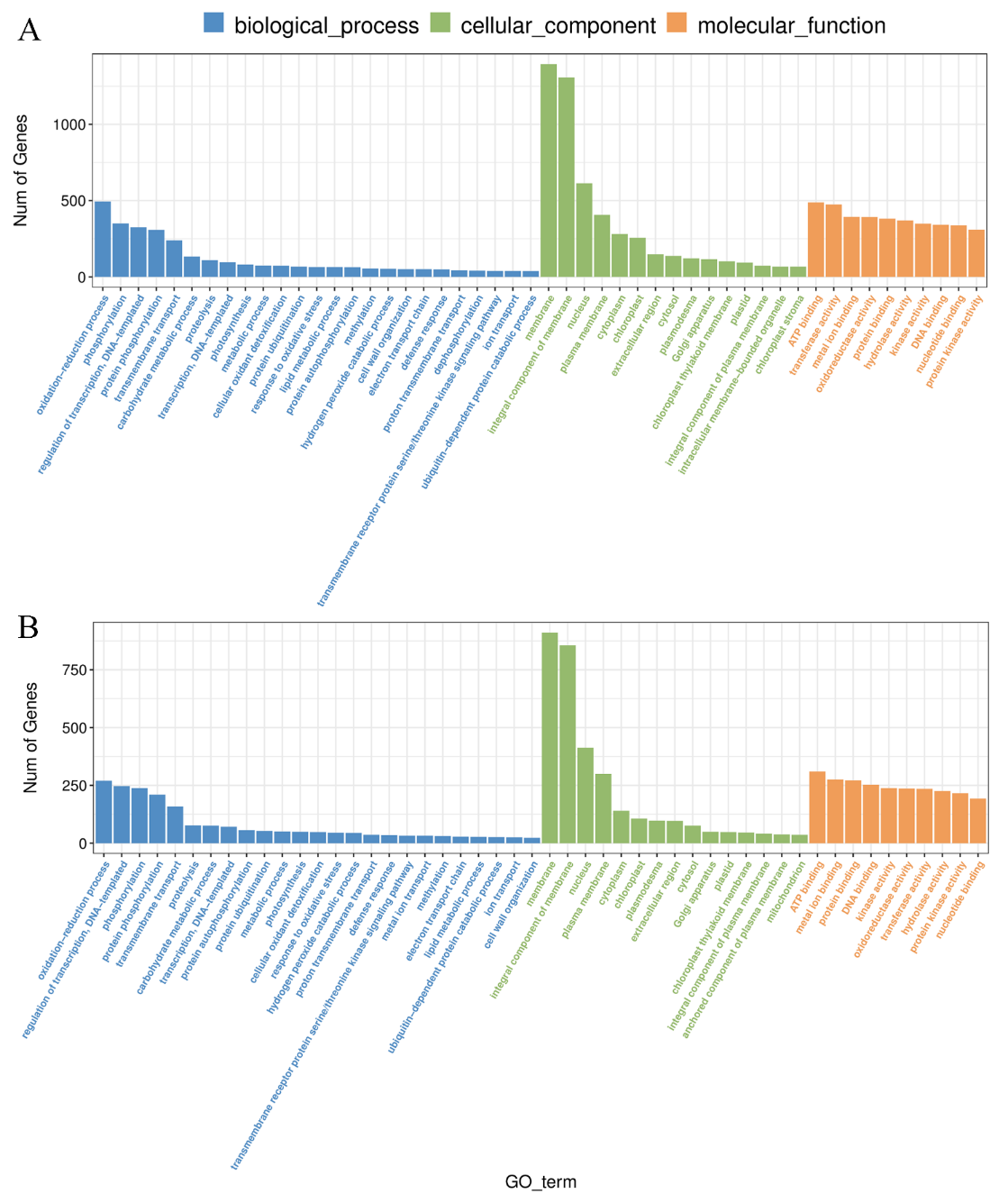


**Figure S1.** GO annotations of DEGs in Qi319 and Zi330 identified under deep sowing treatments. A: QDS *vs.* QCK, B: ZDS *vs*. ZCK.


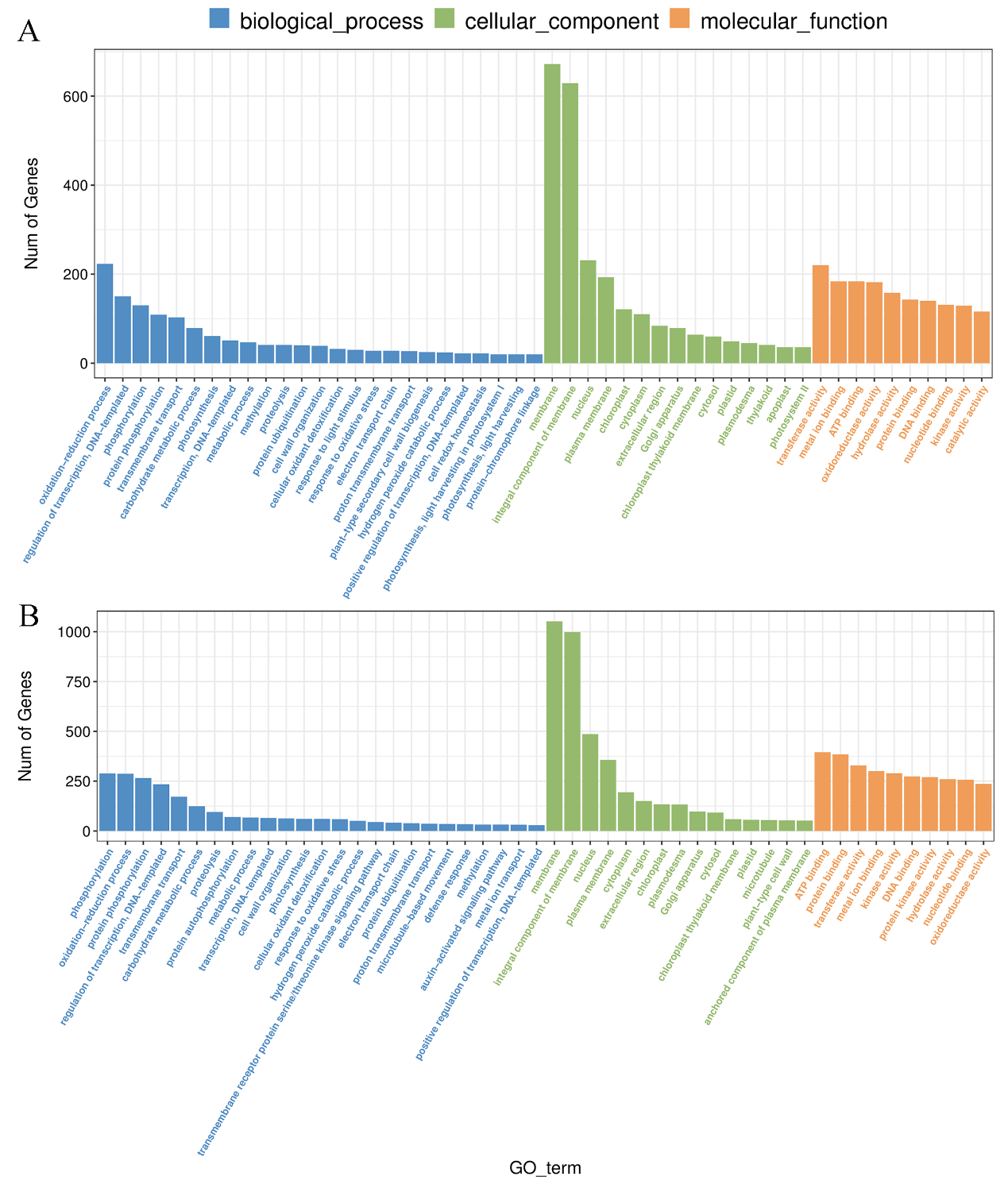


**Figure S2.** GO annotations of DEGs in Qi319 and Zi330 identified after adding exogenous GA under normal sowing depth and deep sowing stress. A: QDSGA *vs.* QCKGA, B: ZDSGA *vs.* ZCKGA.


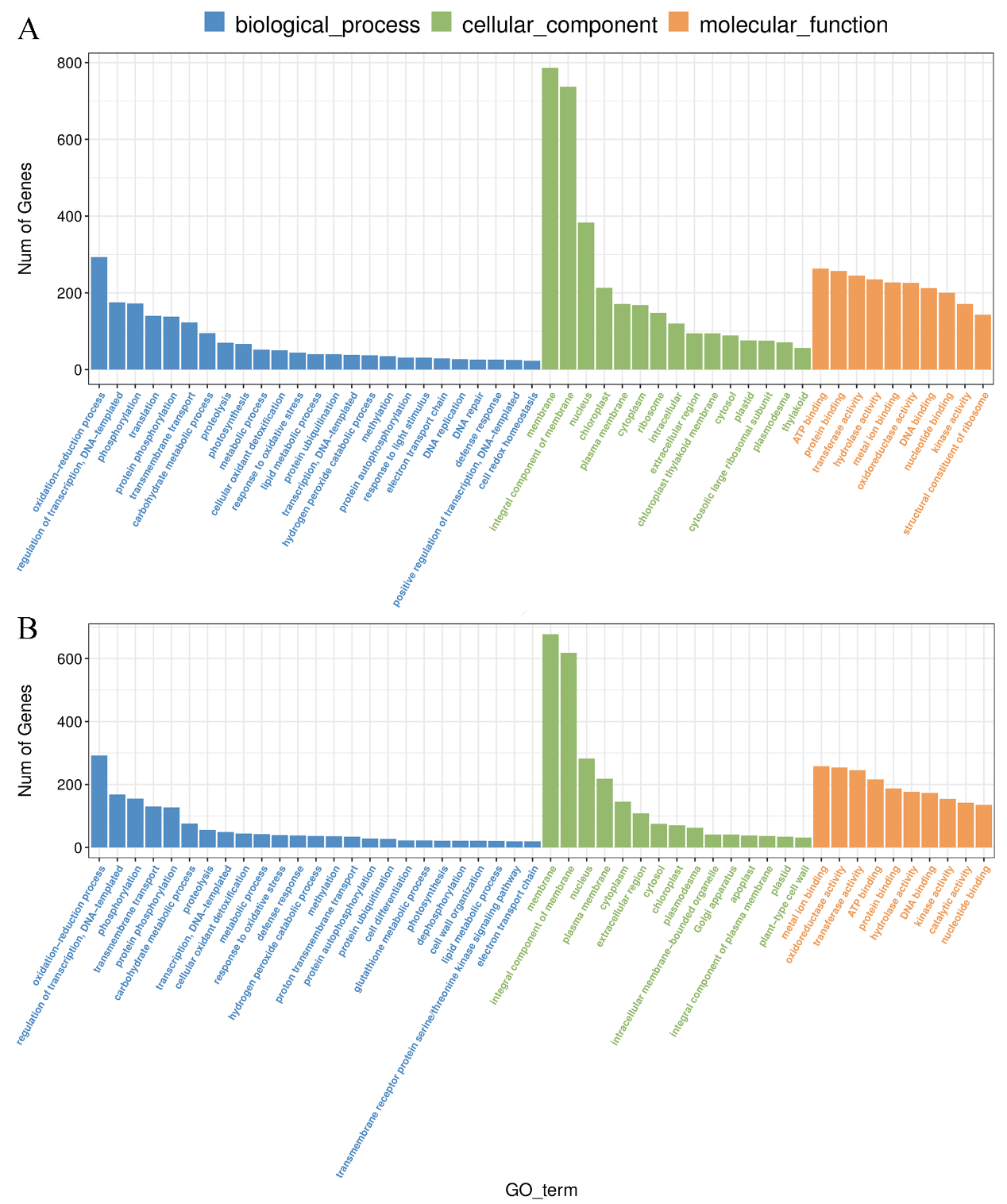


**Figure S3.** GO annotations of DEGs in Qi319 and Zi330 identified after adding exogenous BR under normal sowing depth and deep sowing stress. A: QDSBR *vs.* QCKBR, B: ZDSBR *vs.* ZCKBR.


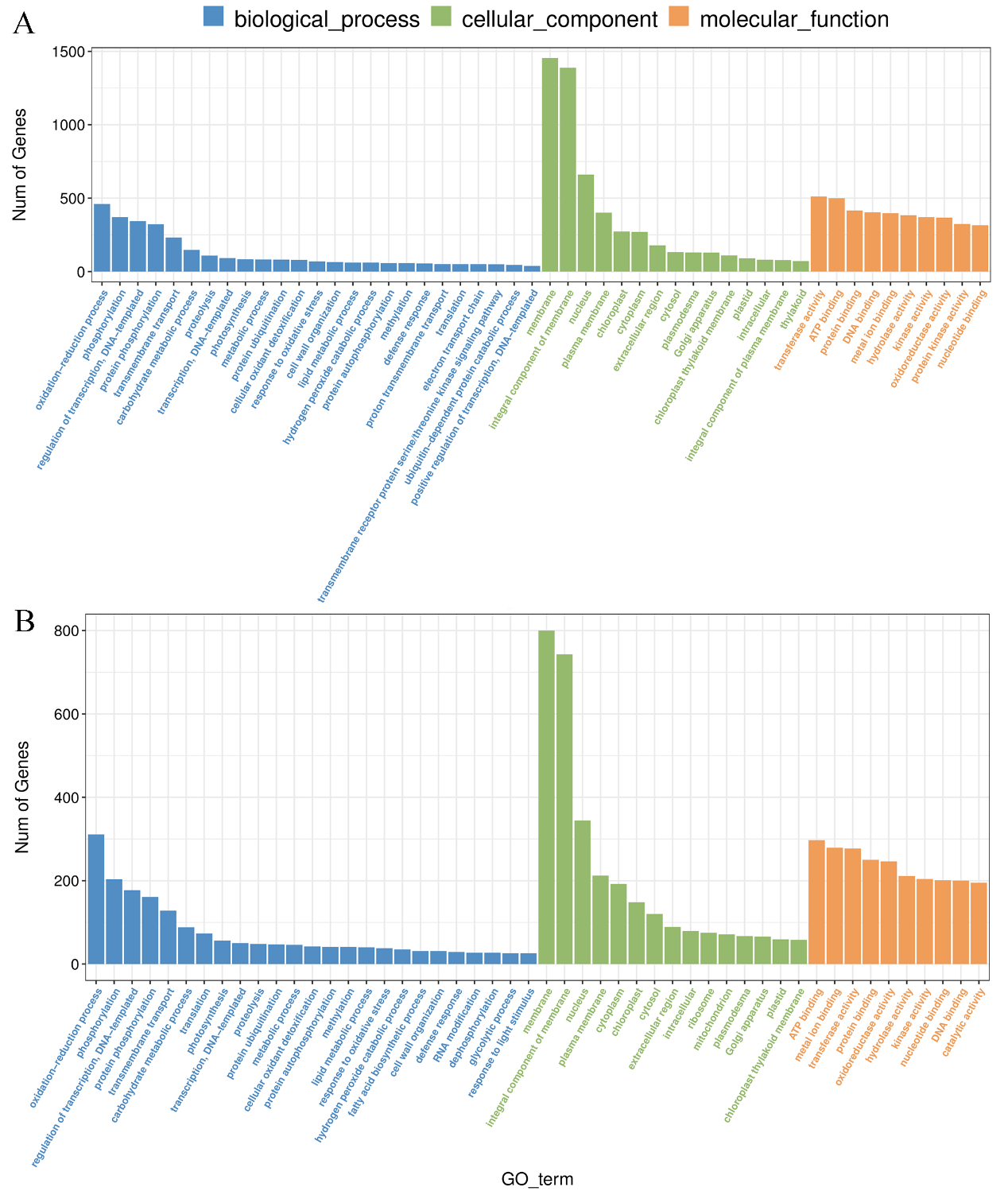


**Figure S4.** GO annotations of DEGs in Qi319 and Zi330 identified after adding exogenous SL under normal sowing depth and deep sowing stress. A: QDSSL *vs.* QCKSL, B: ZDSSL *vs.* ZCKSL.
